# The COMT Val158 Met polymorphism as an associated risk factor for Alzheimer disease and mild cognitive impairment in APOE 4 carriers

**DOI:** 10.1186/1471-2202-10-125

**Published:** 2009-09-30

**Authors:** Manuel Fernández Martínez, Xabier Elcoroaristizabal Martín, Luís Galdos Alcelay, Jessica Castro Flores, Juan María Uterga Valiente, Begoña Indakoetxea Juanbeltz, María Ángeles Gómez Beldarraín, Josefa Moraza López, María Carmen Gonzalez-Fernández, Ana Molano Salazar, Rocio Bereincua Gandarias, Sandra Inglés Borda, Nuria Ortiz Marqués, Miryam Barandiarán Amillano, María Carrasco Zabaleta, Marian M de Pancorbo

**Affiliations:** 1 Neurology Department, Hospital de Cruces, Baracaldo, Vizcaya, Spain; 2 Research General Service, Bank of DNA and Dpt. of Z. and Cellular Biology, Faculty of Pharmacy, University of Basque Country UPV/EHU, Vitoria-Gasteiz, Álava, Spain; 3 Neurology Department, Hospital de Txagorritxu, Vitoria-Gasteiz, Álava, Spain; 4 Neurology Deparment, Hospital de Basurto, Bilbao, Vizcaya, Spain; 5 Neurology Department, Hospital Donosti, San Sebastian, Guipuzcoa, Spain; 6 Neurology Department, Hospital de Galdakao, Galdakao, Vizcaya, Spain; 7 Neurology Department, Hospital Santiago Apóstol, Vitoria-Gazteiz, Álava, Spain

## Abstract

**Background:**

The aim of this study is to examine the influence of the *catechol-O-methyltranferase (COMT) *gene (polymorphism Val158 Met) as a risk factor for Alzheimer's disease (AD) and mild cognitive impairment of amnesic type (MCI), and its synergistic effect with the *apolipoprotein E gene (APOE)*.

A total of 223 MCI patients, 345 AD and 253 healthy controls were analyzed. Clinical criteria and neuropsychological tests were used to establish diagnostic groups.

The DNA Bank of the University of the Basque Country (UPV-EHU) (Spain) determined *COMT *Val158 Met and *APOE *genotypes using real time polymerase chain reaction (rtPCR) and polymerase chain reaction (PCR), and restriction fragment length polymorphism (RFLPs), respectively. Multinomial logistic regression models were used to determine the risk of AD and MCI.

**Results:**

Neither *COMT *alleles nor genotypes were independent risk factors for AD or MCI. The high activity genotypes (GG and AG) showed a synergistic effect with *APOE ε4 *allele, increasing the risk of AD (OR = 5.96, 95%CI 2.74-12.94, p < 0.001 and OR = 6.71, 95%CI 3.36-13.41, p < 0.001 respectivily). In AD patients this effect was greater in women.

In MCI patients such as synergistic effect was only found between AG and *APOE ε4 *allele (OR = 3.21 95%CI 1.56-6.63, p = 0.02) and was greater in men (OR = 5.88 95%CI 1.69-20.42, p < 0.01).

**Conclusion:**

*COMT *(Val158 Met) polymorphism is not an independent risk factor for AD or MCI, but shows a synergistic effect with *APOE ε4 *allele that proves greater in women with AD.

## Background

Genetic, metabolic and environmental factors play a role in Alzheimer's disease (AD). The *APOE *ε4 allele is the strongest genetic risk factor for sporadic forms of AD [1]. However, the *APOE *gene explains only a fraction of the genetic risk associated with AD, and it is possible that other genes or metabolic factors may modify the *APOE *ε4 effect to initiate the pathogenesis of AD. One of these candidate genes is the *COMT *(Catechol-O methyltransferase) gene [2].

*COMT *is located on chromosome 22. It has 6 exons, spans 27 kb and encodes a protein of 271 amino acids. There is a single functional nucleotide polymorphism (SNP) on exon 4 of the *COMT *gene: rs4680. This SNP is characterised by low allele A activity (ATG/methionine) and a (high activity) allele G (GTG/valine) in codon 158. O-methylation mediation by *COMT *is an important mechanism for inactivating estrogen. In contrast to the G (high activity) allele, the A (low activity) allele causes the accumulation of catecholestrogens [3].

In the last few years it has been suggested that estrogens could be implicated in the etiology of AD through a *APOE-*dependent mechanism. Estrogen effects upon the central nervous system (CNS) are modulated via the estrogen receptors and metabolites. In mice, estradiol promotes synaptic sprouting in response to an entorhinal cortex lesion model of AD via an APOE-dependent mechanism [4]. Other evidences from animal studies also indicates that 17-β-estradiol exerts a strong protective effect by reducing neuronal apoptosis and inflammatory reponses [5]. 17-β-estradiol activates protein kinase C (PKC) in rat neuron cultures, and this activation is an important step in estrogen protection against β-amyloid [6].

The important decrease in endogenous estrogens levels after menopause may contribute to the development of AD [7]. Data from population-based studies support the hypothesis that estrogen replacement therapy is associated with a reduced prevalence of AD in postmenopausal women [8,9]. These data suggest a protective effect of estrogens upon AD.

Therefore, it is probable that genes such as *COMT*, involved in estrogen metabolism are associated with a greater likelihood of developing AD, and constitute genetic risk markers.

Several studies have examined the relationships between *COMT *polymorphisms and the risk of sporadic AD. Wang et al. (2005) [10] found that *COMT *GG genotype and *APOE *ε4 allele to exert a synergistic effect upon the risk of AD. In addition, Borroni et al. [11] and Sweet et al. [12] have determined that *COMT *genetic variation was associated with a risk of psychosis in AD.

The aim of the present study was to determine whether *COMT *is linked to the risk of AD; whether there is an interaction with *APOE*; and whether such interaction could influence the risk of AD. Our hypothesis is that the effect of *COMT *activity on estrogen levels may exist only between high activity alleles of *COMT *(G alleles) and ε4 status carriers. Accordingly, we have studied this effect in AD patients and in MCI patients, the latter condition possibly representing a prodrome for dementia [13].

With the purpose of examining the influence of *COMT*, a gene involved in estrogen metabolism, as a genetic risk factor for cognitive impairment, we conducted a study of a sample of patients with mild cognitive impairment (MCI), Alzheimer's disease (AD) and a control group. All subjets were analyzed for *COMT *valine/methionine (rs4680) polymorphism and *APOE *genotype.

## Methods

This cross-sectional study comprised three subjects groups: MCI patients (n = 223), AD patients (n = 345) and healthy controls (n = 253). Subjets were prospectively recruited from the Neurology Departments of several hospitals. Participants were aged 50 years and older.

The subjects were evaluated using a broad battery of neuropsychological tests: Minimental State Examination (MMSE), Clinical Dementia Rating scale (CDR), CERAD protocol, Stroop test, unilateral and bilateral motor praxis, 7-minute test, trial making part A and B; and Neuropsychiatric Inventory (NPI). Clinical criteria for dementia and AD (DSM IV and NINCDS-ADRDA) [14,15] and Petersen's criteria for MCI, were used [13,16]. For AD and MCI patients, evaluation also included routine blood tests: haematology, biochemistry, thyroid-stimulating hormone, vitamin B_12 _levels, syphilis serology and neuroimaging test (CT scan or MRI).

Based upon the results of these evaluations, the participants were classified into the following groups: MCI patients, AD patients, and healthy control subjects.

A specific database was designed using Microsoft Access 2002 and declared to the Spanish Data Protection Agency. The study was approved by the Ethics Committee of Cruces Hospital. All patients signed informed consent to undergo the examination. The study was conducted in accordance with the Declaration of Helsinki concerning medical research in human subjects.

### MCI patients

The diagnosis was based on Petersen's criteria. Patients had memory complaints corroborated by an informant, representing a decline from a previous level of functioning given their age and educational level. The score in CDR scale was required to be 0.5, and performance in relation to other cognitive functions and daily living activities were required to be normal.

### AD patients

The diagnosis of AD was based on the DSM IV [14] and NINCDS-ADRDA [15] criteria for probable and possible AD. Patients with a total score of less than 3 on CDR scale (mild to moderate dementia) were included.

### Healthy control subjects

These subjects scored within the normal ranges for age and educational level in psychometric testing, with a CDR score of 0.

The exclusion criteria included: previous cerebrovascular diseases (transient ischemic attacks, stroke or intracranial haemorrhage), other neurodegenerative diseases, severe comorbidities making adequate follow-up unlikely, acute psychiatric diseases, and the absence of a reliable informant.

### Genetic analysis

On the first visit, peripherical blood samples were collected at EDTA vacuum tubes from all individuals. Genomic DNA was extracted from white blood cells using standard phenol/chloroform extraction method. Then *COMT *Val158 Met (rs4680) and *APOE *genotypes were analyzed blinded to clinical diagnosis.

*APOE *was amplified by PCR with the primers 112F and 158R, under the PCR conditions described by Wilton and Lim [17]. Digestion of the amplified product was carried out with *Hae *II and *Afl *III, as described by Álvarez-Álvarez et al. [18]. *COMT *genotyping was done using 5'-nuclease allelic discrimination assay on the ABI Prism 7000 Sequence Detection System. Taqman SNP genotyping products and Custom Taqman SNP Genotyping Assays were used. Reactions were done with the following protocol: 95°C for 10 min, 45 cycles at 95°C for 15 sec, and 58°C for 1 min 30 sec.

### Statistical analysis

Genepop software version 4.0 was used to calculate genotypic and allelic frequencies and to test the goodness of fit of patients and control samples to Hardy-Weinberg equilibrium by means of the exact test of Guo and Thompson [19].

Statistical analysis was also performed using the SPSS^® ^package, version 15.0. Differences among demographic and clinical variables were evaluated using the chi- squared test and analysis of variance (ANOVA). A dichotomous variable was used for each polymorphism: "yes" or "no" for "carrier" or "no carrier" of the *APOE ε4 *allele and for different alleles and genotypes from the *COMT *gene.

A multinonial regression model was created in order to determine the independent effect of any *COMT *allele and genotypes in the absence of ε4 allele; and the effect of ε4 allele in the total sample and a sample selected by at least one ε4 allele and no G *COMT *allele. Another model was created to evaluate the combined effect of ε4 allele and *COMT *genotypes, based on the hypothesis that the effect of estrogens might exists only in ε4 carriers. In all these models the reference categories were controls, females and the absence of ε4 and G *COMT *allele. P-values of less than 0.05 were considered statistically significant.

## Results

We have investigated the independent and combined association of *COMT *Met108/158Val and *APOE *using a case-control design.

In the present study we analyzed a sample of 223 MCI patients, 345 AD patients, and 253 healthy control subjects without significant differences in terms of age (p > 0.05).

The MMSE scores for MCI, AD patients and controls were 26.58 ± 2.16, 19.72 ± 4.80 and 28.23 ± 1.73 respectively (p < 0.05) (Table 1).

**Table 1:** Baseline demographics.

**Group**	**Age^a^**	**Women (%)^b^**	**MMSE^c^**
MCI	74.10 ± 8.81	60.3	26.58 ± 2.16

AD	74.95 ± 7.83	71.4	19.72 ± 4.80

CONTROLS	74.94 ± 10.34	59.6	28.23 ± 1.73

^a ^Years, mean ± standard deviation (SD). ^b ^% of women in group. ^c ^MMSE score, mean ± standard deviation.

Table 2 shows the allele and genotype frequencies of *COMT *and *APOE *genes in MCI, AD and controls. In all studied groups, the *COMT *genotype frequencies were in Hardy-Weinberg equilibrium (p > 0.05). There were no significant differences in allele and genotype frequencies in MCI and AD compared to controls for *COMT *gene, while the differences proved significant for *APOE *gene (Table 3).

**Table 2:** Allele and genotype frequency.

**COMT**		**MCI^a ^(n = 223)**	**AD^b ^(n = 345)**	**Controls (n = 253)**
Allele	A	0.520	0.470	0.512

	G	0.480	0.530	0.488
	
Genotype	AA	0.278	0.214	0.265

	AG	0.484	0.510	0.494
	
	GG	0.238	0.275	0.241
	
H-W^a^	p-Value	0.69	0.75	0.90

APOE		MCI^a ^(n = 223)	AD^b ^(n = 345)	Controls (n = 253)

Allele	2	0.027	0.035	0.059

	3	0.731	0.664	0.838
	
	4	0.242	0.301	0.103
	
Genotype	2.2	0.000	0.000	0.008

	2.3	0.045	0.052	0.095
	
	2.4	0.009	0.017	0.008
	
	3.3	0.561	0.432	0.692
	
	3.4	0.296	0.412	0.198
	
	4.4	0.090	0.087	0.000
	
H-W^c^	p-value	0.07	0.85	0.11

^a^MCI: mild cognitive impairment. ^b^AD: Alzheimer's disease. ^c^Hardy-Weinberg probability test.

**Table 3:** Exact G test, allele and genotype frequencies.

**COMT^a^**			**COMT^b^**		
	
	**p-value**	**S.E**.^c^		**p-value**	**SE^c^**
MCI vs controls	0.85	<0.001	MCI vs controls	0.85	<0.001

MCI vs AD	0.11	<0.001	MCI vs AD	0.10	<0.001

AD vs controls	0.16	<0.001	AD vs controls	0.16	<0.001

**APOE^a^**			**APOE^b^**		

	**p-value**	**SE^c^**		**p-value**	**SE^c^**
	
MCI vs controls	0.00	<0.001	MCI vs controls	0.00	<0.001

MCI vs AD	0.06	<0.001	MCI vs AD	0.06	<0.001

AD vs controls	0.00	<0.001	AD vs controls	0.00	<0.001

^a ^Allele frequency. ^b^Genotype frequency. ^c ^Standard error.

In order to determine whether *COMT *G allele is an independent risk factor for MCI and AD, we selected a subgroup of MCI, AD and control individuals with the presence of at least one *COMT *G (high activity) allele and the absence of *APOE *ε4 allele.

The odds ratios (ORs) of developing MCI and AD were 0.90 (95%CI 0.53-1.51, p < 0.68) and 1.24 (95%CI 0.74-2.08, p = 0.42), respectively (Table 4). In subsequent analyses we obtained that any *COMT *genotype with at least one high activity allele (G) had a significative effect (data not shown).

**Table 4:** Risk factors for MCI and AD. Logistic regression models.

	**MCI**		**AD**	
	
**Independent effects**	**OR IC95%**	**P**	**OR IC95%**	**p**
**G (+) NO E4 (+)^a^**	0.90 (0.53-1.51)	0.68	1.24 (0.74-2.08)	0.42

**E4 (+)^b^**	2.53 (1.69-3.79)	<0.001	4.37 (3.02-6.33)	<0.001

E4 (+)*Women^b1^	2.46 (1.45-4.15)	<0.001	5.10 (3.20-8.13)	<0.001

E4 (+)*Men^b2^	2.59 (1.36-4.92)	0.04	3.19 (1.72-5.91)	<0.001

**E4 (+) NO COMT_G**^b3^	1.64 (0.80-3.36)	0.18	2.61 (1.31-5.18)	<0.01

	**MCI**		**AD**	
	
**Synergistic effects**	**OR IC95%**	**p**	**OR IC95%**	**p**

E4 (+) * GG^c^	2.11 (0.90-4.95)	0.08	5.96 (2.74-12.94)	<0.001

E4 (+) * AG^c^	3.21 (1.56-6.63)	0.02	6.71 (3.36-13.41)	<0.001

E4 (+) * GG * WOMEN^d^	2.00 (0.60-6.67)	0.26	11.50 (3.83-34.53)	<0.001

E4 (+) * GG * MEN^d^	3.30 (0.88-12.35)	0.07	2.14 (0.64-7.20)	0.22

E4 (+) * AG * WOMEN^d^	2.40 (0.96-6.00)	0.06	7.94 (3.24-19.42)	<0.001

E4 (+) * AG * MEN^d^	5.88 (1.69-20.42)	<0.01	5.36 (1.76-16.28)	<0.01

^a ^Sample selected by at least one *COMT *genotype and absence of E4. Reference category was sample control. ^b ^Total sample with any exclusion, control sample was reference. ^b1 ^Total sample with any exclusion, female control sample was reference. ^b2 ^Total sample with any exclusion, male control sample was reference. ^b3^Sample selected by at least one E4 and none *COMT *G allele. Reference category was sample control. ^c ^Sample selected by at least one E4 and *COMT *genotype. ^d ^Sample selected by at least one E4, *COMT *genotype and gender. *In all cases, reference category was control sample.

*APOE *ε4 allele is a risk factor for cognitive impairment, and this risk is lower in MCI (OR = 2.53, 95%CI 1.69-3.79 p < 0.001) than in AD (OR = 4.37, 95%CI 3.02-6.33 p < 0.001). The higher risk conferred by allele *APOE *ε4 was observed even when the samples were subgrouped by sex: MCI women (OR = 2.46, 95%CI 1.45-4.15 p < 0.001) versus AD women (OR = 5.10, 95%CI 3.20-8.13 p < 0.01) and MCI men (OR = 2.59, 95%CI 1.36-4.92 p < 0.04) versus AD men (OR = 3.19, 95%CI 1.72-5.91 p < 0.001).

Aiming to avoid the combined effect of *COMT *genotypes and allele *APOE *ε4, we analyzed the risk of MCI and AD according to the presence of at least one *APOE *allele ε4 and the absence of *COMT *G allele. The OR for MCI and AD were OR = 1.64, 95%CI 0.80-3.36, p = 0.18, and OR = 2.61, 95%CI 1.31-5.18, p < 0.01 respectively. These results could indicate a synergistic interaction between APOE ε4 and *COMT *G in MCI and AD patients.

We further evaluated a possible synergistic effect between *COMT *and *APOE *by using a multivariate logistic regression model. To this effect, we subgrouped the subjets according to the high activity genotypes of *COMT *(GG or AG) and the presence of at least one *APOE *ε4 allele.

A synergistic effect between genotypes GG and AG with *APOE *ε4 was observed in AD patients (OR = 5.96, 95%CI 2.74-12.94, p < 0.001 and OR = 6.71 95%CI 3.36-13.41, p < 0.001 respectively). This effect is most notorious is women for both genotypes GG and AG (OR = 11.50, 95%CI 3.83-34.53, p < 0.001 and OR = 7.94 (95%CI 3.24-19.42, p < 0.01 respectively).

In MCI patients, this synergistic effect was only found between AG and *APOE *ε4 (OR = 3.21 95%CI 1.56-6.63, p = 0.02) and was greater in men (OR = 5.88 95%CI 1.69-20.42, p < 0,01) (Table 4).

## Discussion

Our study shows that neither *COMT *alleles nor genotypes are independently associated with the risk of AD or MCI, but confirms an association between high activity alleles of *COMT *(G alleles), ε4 status carriers and the risk of AD. This effect is moreover greater in AD women.

In our series, *APOE *ε4 allele is seen to be an independent risk factor for the AD population, and this risk is highest for women. The *APOE *ε4 allele also constitutes a risk factor for MCI patients. On evaluating the independent effect of the *APOE *ε4 allele in the absence of *COMT *genotype G, the risk for AD remains; though the association with the risk of MCI is lost.

We have found the GG and AG genotypes to exert a synergetic effect with *APOE *ε4. When these genotypes are included into the multinomial analysis, the risk of AD in *APOE *ε4 allele and GG or AG carriers increases, and is more pronounced in women. The risk for MCI is only found in *APOE *ε4 allele and AG carriers, and is more notorious in men.

To our knowledge, only two previous studies have evaluated the role of *COMT *polymorphism as a risk factor for AD [10,20]. The first study was conducted in Colombia by Forero et al. [20], and an initial association between AA low activity genotype and the risk of AD males (OR = 2.76, 95%CI 1.08-7.08, p = 0.02) was found. However, these associations were lost after Bonferroni correction for multiple testing.

Our findings are in accordance with the second study performed by Wang et al. [10]. These authors established that the GG genotype (*COMT *HH) and the presence of at least one *APOE *ε4 allele exerted a synergetic effect, increasing the risk of AD to 3.6 (95%CI 1.2 - 10.6). They postulated that the existence of the *COMT *HH genotype is a important modulating factor for the increased risk of developing AD. The sample size of our study allows a stratification by sex. In our series, similar ranges of values have been obtained for the most high activity genotypes (AG and GG), and mainly women with AD

There is considerable evidence suggesting that allele ε4 constitutes a major susceptibility factor for the development of AD [1]. Some authors [21] have shown the *APOE *ε4 allele to be associated with an increased risk of MCI (OR: 6.04, 95%CI: 2.76-3.23; p < 0.001), though with no effect upon the probability of evolving AD. Despite the fact that the presence of the *APOE *ε4 allele has been associated with an increased risk of conversion from MCI to AD, the sensitivity is quite low [22]. The *APOE *ε4 allele is an important risk factor for AD, and may be useful for predicting who is likely to progress to dementia [23]. However, *APOE *ε4 carriers do not always develop dementia, and *APOE *ε4 carrier status in itself was not found to be predictive of cognitive decline or conversion to AD from MCI [24]. The role of *APOE *in brain repair mechanisms might be associated with the risk for AD, the ε4 allele being less effective in synaptic remodeling, repair, and regeneration after brain injuries [25].

Several studies have shown an interaction between estrogen and *APOE*. Sex-specific incidence rates for AD are higher in women after menopause than in men [26], probably due to lack of estrogens, and the *APOE *ε4 allele is associated with a greater decline in cognitive functions in women. This finding may be supportive of sex differences in *APOE*-associated risk for AD [27]. Estrogen use was associated with a lesser cognitive decline among ε4-negative women [28].

The biological mechanisms underlying the effect of *COMT *gene upon the risk of AD fall beyond the scope of the present study, though different hypotheses could be postulated based on the literature. Worda et al. 2003 [29] showed that women with an high activity genotype (GG) present lower serum estrogen concentration. Therefore, this genotype could be correlated to a lesser neuroprotective effect, and differences in *COMT *genotype might be involved in causing variable effects of estrogens upon diseases. 17-β-estradiol is metabolized via two major pathways: 16-alpha-hydroxilation or the formation of cathecholestrogens [30]. *COMT *enzyme participates in the metabolism of estrogens after their hydroxylation to catecholestrogens by forming O-methyl derivates [3]. Thus, polymorphism in the *COMT *gene affecting the activity of the enzyme could alter the levels of cathecolestrogens and consequently the estrogen neuroprotective effect.

The ways in which *COMT *overactivity may interact with *APOE *ε4 comprise the lowering of brain estrogen levels.

Tau hyperphosphorylation is proposed to be an early event characteristic of AD and other neurodegenerative diseases [31]. The neurofibrillary tangles present in AD consist mainly of hyperphosphorylated microtubule-associated protein tau [32]. 17 β-estradiol exerts a neuroprotective effect through the inactivation of GSK-3β, which is the kinase most implicated in tau hiperphosphorylation [33].

Another possible mechanism of action of estrogen is related to oxidative stress. Eichi et al. [34] demonstrated that catecholestrogens were catechol metabolites of 17-β estradiol that induced DNA adducts. Oxidative damage may be an early event in the pathogenesis of AD when the balance between free radical generation and antioxidant capacities shifts toward free radical production [35]. This fact is already evident in early AD [36]. Finally, excessive *COMT *activity may induce saturation of methylation capacity, deficient methylation capacity having been linked to neurodegeneration. The metabolism of levodopa down the *COMT *pathway has been speculated to result in the saturation of cellular methylation capacity in Parkinson's disease (PD) and in accelerated cognitive decline and dementia [37].

The distinction between *APOE *ε4 carriers and non-carriers in AD and MCI appears to be increasing in importance: in MCI, the risk of cognitive decline and progression to AD seems to be greatest in individuals who carry at least one copy of both the BCHE-K and *APOE *ε4 alleles. In a recent paper [37], *APOE *ε4 and butyrylcholinesterase-K (BuChE-K) were associated with an increased risk for cognitive decline in patients with Parkinson's Disease Dementia (PDD) [38]

Pharmacogenomics and the study of interindividual genetic variability, which plays a significant role in defining drug response and toxicity, could exert a potential influence upon the design of more efficacious and safe treatments. Rosiglitazone [39] and passive antibodies targeted to beta-amyloid peptide [40] have elicited treatment response in non-ε4 carriers with mild to moderate AD.

Some limitations to our study must be adressed. The main limitation is that the study population comes from the hospital setting. A community-based study could provide more information. The serum leves of estradiol have not been measured, and we do not know whether the patients received estrogen replacement therapy in the last years. We also include a sample of patients with MCI, which is probably a heterogeneous clinical entity. Not all the subjects in this group develop AD, and some of them return to a normal cognitive status. More operational criteria for this clinical entity are nedeed. Such a lack of operational criteria could explain our discordant results in this particular population (more risk for men than women). Accordingly, prospective studies might elucidate not only the role of *COMT *genotypes in AD, but also in the evolution of cognitive impairment from the early stages.

Our study also may be biased by the fact that the recruited MCI subjects could be in different stages of the disease, and individuals with a increased *COMT *activity genotype and *APOE *ε4 carrier status can convert more rapidly to AD, such individuals being under-represented in the MCI sample. This may explain the lower risk associated with the GG versus the AG genotype in MCI ε4 carriers.

The strengths of our study are its multicenter nature including AD patients, healthy controls, and for the first time MCI patients. Moreover, the patient sample is not small, and the number of cases allows gender stratification.

The activity of the main enzymes implicated in acetylcholine metabolims (acetylcholine esterase and choline acetyltranferase) is affected by estrogen administration [41,42]. Based on this interaction between *APOE *and *COMT *genotypes, it could be possible to identify responder patients, that could derive more benefit from acetylcholinesterase inhibitors, actually the main treatment for AD. If the interaction between *APOE *ε4 and *COMT *genotypes in patients with AD is confirmed by future studies, new drugs could be developed to treat AD by regulating synaptic dysfunction and estrogen metabolism. Treatments that reduce *COMT *activity might prove useful in the treatment of AD or in preventing the progression from MCI to AD, especially in women. Elucidation of the mechanisms whereby increased *COMT *activity influences neurodegenerative processes in ε4 carriers might help clarify etiological mechanisms in AD.

## Conclusion

In conclusion, *COMT *(Val158 Met) polymorphism is not an independent risk factor for AD or MCI, but exerts a synergistic effect with the *APOE *ε4 allele which is greater in women with AD. In MCI patients, this synergistic effect was only found between AG and *APOE *ε4, and was greater in men.

## List of abbreviations

AD: Alzheimer's disease; APOE: *apolipoprotein E gene*; COMT: *Catechol-O-methyltranferase gene*; MCI: Mild cognitive impairment of amnesic type.

## Competing interests

The authors declare that they have no competing interests.

## Authors' contributions

MFM: main investigator, conceived of the study, and participated in its design and coordination, and drafted the manuscript. XEM: co-investigator; participated in its design and coordination, and drafted the manuscript. JCF: co-investigator; participated in its design and coordination, and drafted the manuscript. LGA: co-investigator; participated in its design and coordination, and drafted the manuscript. JMUV: co-investigator; participated in its design and coordination. BIJ: co-investigator; participated in its design and coordination. MAGB: co-investigator; participated in its design and coordination. JML: co-investigator; participated in its design and coordination. MCGF: participated in genetic analysis. AMS: performed the battery of neuropsychological tests. RBG: performed the battery of neuropsychological tests. SIB: performed the battery of neuropsychological tests. NO: performed the battery of neuropsychological tests. MBA: performed the battery of neuropsychological tests. MCZ: performed the battery of neuropsychological tests. MMP: co-investigator; participated in its design and coordination, and drafted the manuscript. All authors read and approved the final manuscript.
